# Age-related fornix decline predicts conservative response strategy-based slowing in perceptual decision-making

**DOI:** 10.1016/j.nbas.2024.100106

**Published:** 2024-01-24

**Authors:** Lauren Revie, Claudia Metzler-Baddeley

**Affiliations:** Cardiff University Brain Research Imaging Centre (CUBRIC), School of Psychology, Cardiff University, Maindy Road, Cardiff CF24 4HQ, United Kingdom

**Keywords:** Aging, Visuo-perceptual decision-making, Speed-accuracy trade-off, Drift diffusion model, Fornix, Magnetic resonance spectroscopy, Magnetic resonance imaging, Multishell diffusion weighted imaging

## Abstract

Aging leads to response slowing but the underpinning cognitive and neural mechanisms remain elusive. We modelled older and younger adults’ response times (RT) from a flanker task with a diffusion drift model (DDM) and employed diffusion-weighted magnetic resonance imaging and spectroscopy to study neurobiological predictors of DDM components (drift-rate, boundary separation, non-decision time). Microstructural indices were derived from white matter pathways involved in visuo-perceptual and attention processing [optic radiation, inferior and superior longitudinal fasciculi (ILF, SLF), fornix]. Estimates of metabolite concentrations [N-acetyl aspartate (NAA), glutamate (Glx), and γ-aminobutyric acid (GABA), creatine (Cr), choline (Cho), myoinositol (mI)] were measured from occipital (OCC), anterior cingulate (ACC) and posterior parietal cortices (PPC). Age-related increases in RT, boundary separation, and non-decision time were observed with response conservatism acounting for RT slowing. Aging was associated with reductions in white matter microstructure (lower fractional anisotropy and restricted signal fraction, larger diffusivities) and in metabolites (NAA in ACC and PPC, Glx in ACC). Regression analyses identified brain regions involved in top-down (fornix, SLF, ACC, PPC) and bottom-up (ILF, optic radiation OCC) processing as predictors for DDM parameters and RT. Fornix FA was the strongest predictor for increases in boundary separation (beta = −0.8) and mediated the effects of age on RT. These findings demonstrate that response slowing in visual discrimination is driven by the adoption of a more conservative response strategy. Age-related fornix decline may result in noisier communication of contextual information from the hippocampus to anterior decision-making regions and thus contribute to the conservative response strategy shift.

## Introduction

One of the best-established findings in aging research concerns the slowing of response speed [79] that can be observed in a wide range of tasks involving simple decision-making to more complex executive functioning with spatial-perceptual discrimination being disproportionally affected [72], [94].

Age-related slowing is often accompanied by a lengthening of the speed accuracy trade-off (SAT) which refers to the trade-off that occurs between responding as timely and as accurately as possible when completing a time-pressured cognitive task. Greater SATs are thought to occur due to older adults adopting a more cautious response strategy that favours response accuracy over speed [86]. In contrast, younger adults typically make faster responses which may be at greater risk of errors [17]. It is commonly thought that the change to a more cautious response strategy at the cost of slower reaction times (RT) arises due to an age-related decline in sensorimotor functions leading to a distrust in being able to provide a correct response.

Impairments in sensorimotor functions may affect both bottom-up sensory and top-down decision-making and motor execution processes. According to the sensory degradation hypothesis [35], [102], age-related deterioration in sensory functions results in noisier sensory input and hence longer perceptual processing time for effectively interpreting a stimulus. This in turn increases overall RTs or the likelihood of an incorrect response if RT is not lengthened [4]. Indeed, age-related perceptual decline has been observed at some of the lowest levels of visual processing such as visual contrast, even in those with intact visual acuity and an absence of visual impairment [22], [29].

In addition, the “slowed motor response” hypothesis proposes that age-related increases in RTs emerge from a slowing of top-down decision-making and motor generation and execution processes [5], [24]. This view is backed up by evidence from electroencephalogram studies suggesting that older people are most compromised at the interface of translating a stimulus input into a response output [5], [38].

It is plausible that both sensory degradation and motor noise contribute to a slowing of decision-making processes in aging. However, the analysis of overall RTs or SATs alone, does not allow for a separation of the distinct cognitive components that may contribute to age-related slowing.

Sequential-sampling models, such as Ratcliff’s drift diffusion model (DDM) [70], [71], [72], can be applied to RT data from choice RT tasks to estimate parameters that map distinct cognitive components involved in decision making. The DDM approach has been employed by several studies to clarify the cognitive underpinnings of older adults’ slowed RTs and SATs (see for review Theissen et al., 2021).

**Fig. 1:**
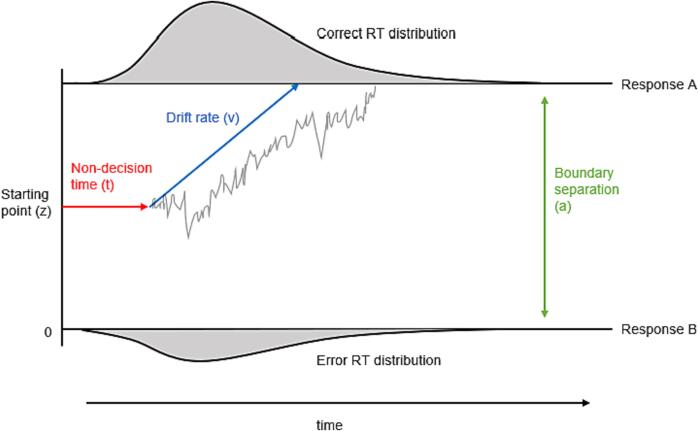
The drift diffusion model (DDM) of response time. Upper and lower lines ‘Response A’ (correct) and ‘Response B’ (incorrect) denote the different responses in a 2-choice task (for example, left or right key press). Reaction times (RT) are fit to the DDM to return an estimate of non-decision time (t) (red) reflecting perceptual and motor processing time, boundary separation value (a) (green), reflecting the distance between the two response criterion thresholds for A or B that reflects the amount of information that needs to be accumulated to trigger a response, and drift rate (v) (blue) reflecting the efficiency of the drift process. Information is thereby assumed to accumulate in a random walk-like diffusion process (gray wiggly line) that commences at the starting point toward one of the two response boundaries. Adapted from [39]. (For interpretation of the references to colour in this figure legend, the reader is referred to the web version of this article.)

According to the DDM, sensory input provides information that accumulates over time. This information fluctuates randomly between two thresholds: a lower threshold representing an incorrect response choice and an upper threshold representing the correct response choice (Fig. 1). When the accumulated information, after some time, crosses one of these thresholds, it triggers the corresponding response. The main components of the DDM, that control the time it takes to reach one of these thresholds include the speed of information uptake (drift rate) (Fig. 1 blue), the distance between the thresholds, that reflects the degree of conservatism regarding the response criterion (boundary separation) (Fig. 1 green), and the time required for non-decisional processes including sensory-perceptual encoding and motor response execution (non-decision time) (Fig. 1 red). If boundary separation decreases (while keeping drift rate non-decision time constant), the response time becomes shorter, but the likelihood of making an error increases. Conversely, if boundary separation increases (again with drift rate and non-decision time held constant), the response times lengthens, but the likelihood of making an error decreases. Thus, according to Ratcliff’s DDM model, the distance between the thresholds, i.e., the boundary separation value, determines the length of the SAT.

Indeed, in the literature higher boundary separation values and longer non-decision times have been reported consistently in older compared with younger individuals while differences in drift rates were found to be moderated by task type (e.g. episodic versus semantic memory) and difficulty [88], [68].

To date only a few studies have investigated the neurobiological underpinnings of age-related differences in DDM parameters, [26], [39], [48], [56], [101]. Magnetic resonance imaging (MRI) studies have found age-related increases in boundary separation to be associated with reduced striatal activity [as measured with the blood oxygen level dependent (BOLD) signal] [39] and with reduction in fractional anisotropy (a diffusion tensor imaging (DTI) based measurement of fiber directionality/coherence) in white matter connections between the striatum and the pre-Supplementary Motor Area (preSMA) [26]. Other studies have linked age-related increases in non-decision time [47], [48] and in drift rate [51] to differences in the BOLD signal in fronto-parietal regions. These findings are consistent with evidence suggesting that reductions in fronto-parietal activity may underpin age-related slowing in simple and choice RT tasks and SAT [8], [36], [45], [56]. They also accord with well-documented evidence of structural and functional changes in fronto-parietal and striatal networks with age [10], [59], [73] that are thought to be important for attention control and decision making [44], [50]. In summary, the findings from DDM based studies suggest that age-related increases in RTs and SAT may be driven by non-decision related sensorimotor decline (non-decision time) and by longer processing times required before a decision threshold can be reached (boundary separation). They further suggest that age-related neural decline in fronto-parietal and striatal decision-making networks contribute to the differences in DDM components in aging. However, the nature of the contribution of age-related changes in visual-attention networks to increases in DDM parameters and response slowing remain elusive.

The aim of the present study was to further elucidate the cognitive and neurobiological substrates of age-related slowing in visual-perceptual discrimination using an Eriksen 2-choice flanker test [23]. The Eriksen flanker task is a well-established response conflict paradigm that allows the modelling of RTs with DDM and has been shown to rely on prefrontal cortex and striatal decision-making regions [87]. The task involves the presentation of a target arrow flanked by distractor arrows, which are either congruent with the directional response to the target, i.e., a left or right key press, incongruent (pointing into the opposite direction), or neutral. Younger (n = 25, age range = 18–29 years) and older participants’ (n = 25, age range = 62–80 years) RT data from the flanker task were modelled to derive SAT and DDM parameters. Correlation coefficients between RTs, SAT, and DDM-derived parameters were calculated and mediation analysis [33] was used to identify those DDM component(s) that accounted for the shared variation in RTs and SAT across both groups. Gray matter metabolic and white matter microstructural measurements were derived from key regions of interest (ROIs) within visual-perceptual and decision-making networks and hierarchical regression analyses were conducted to identify neurobiological brain predictors of cognitive components.

For this purpose, we employed multi-shell high angular resolution diffusion imaging (msHARDI) [19] to quantify white matter microstructural properties and magnetic resonance spectroscopy (MRS) to measure gray matter metabolite concentrations. These modalities were chosen because it is well established that microstructural properties of white matter brain connections, that allow the efficient communication within and between brain networks, deteriorate with advancing age and contribute significantly to cognitive decline including response slowing in aging [40]. Most studies that have investigated age effects on white matter microstructure employed diffusion tensor imaging (DTI) and have consistently found reduced fractional anisotropy (FA) and increased mean diffusivity (MD), axial diffusivity (AD) and radial diffusivity (RD) in older relative to younger participants [9], [14]. These age-related differences in white matter microstructure occur across the whole brain but are particularly apparent in fronto-parietal and limbic regions [11] and correlate with age-related differences in processing speed, episodic memory, and executive functions [37], [40].

Here we studied the microstructure of the following white matter pathways that connect visual and attention network regions and are known to be involved in top-down and/or bottom-up visual perceptual processing and attentional functioning: the optic radiation, that connects the lateral geniculate nucleus with the primary visual cortex in the occipital lobe and is important for bottom-up visual sensory processing [81]; the inferior longitudinal fasciculus (ILF), that connects occipital and anterior temporal cortices and is involved in bottom-up visual-perceptual object, face, and place processing; the fornix, the main output tract of the hippocampus to other limbic and cortical regions, that mediates mnemonic and complex visual discrimination functions [41]; and the superior longitudinal fasciculus (SLF), that connects parietal with prefrontal cortices, notably the right SLF being the crucial white matter pathway of the top-down right-lateralized attention-executive network [89]. White matter microstructural properties of these tracts were not only characterised with DTI metrics (FA, MD, RD, AD) but also with the restricted signal fraction FR, a proxy index for axonal density, from the Composite Hindered and Restricted Model of Diffusion (CHARMED) [2]. FR is thought to be a valuable metric to quantify in this context, as it has been shown to be more sensitive than DTI indices and has been suggested as a potential biomarker for axonal microstructure changes [16] which are well-established in aging.

Furthermore, aging is also known to be associated with changes in concentrations of metabolites that are important for healthy neuronal functioning. More specifically, older compared with younger adults show reduced concentrations of N-acetyl aspartate (NAA) [43], an estimate of neuronal density and function, and of glutamate/glutamine (Glx) and γ- aminobutyric acid (GABA), the major excitatory and inhibitory neurotransmitters in the brain [69], [85]. In addition, aging has been found to be associated with increased concentrations of creatine (Cr), choline (Cho) and myoinositol (mI), that have been linked to inflammation, demyelination, and glia cell proliferation [28], [103]. Such age-related differences in metabolites have been observed in many brain regions including the occipital, frontal, and anterior and posterior cingulate cortices [12], [31], [32], [27], [49], [64], [66], [82]*.* Here we measured concentrations of NAA, Glx, GABA, creatine, choline and mI in the following three cortical ROIs: the occipital cortex (OCC), the anterior cingulate cortex (ACC) and the posterior parietal cortex (PPC). These ROIs were selected as they form key regions of visual perception and attention networks that mediate bottom-up and top-down processing streams with OCC being involved in primary visual processing [64], PPC mediating sensory-perceptual integration [12] and ACC playing a key-role in decision-making by means of error signalling and event rewarding [98].

In this way we were able to characterise age-related metabolic and microstructural differences in key structures of the visual and attentional networks and assess whether these brain differences were predictive of differences in RT, SAT, and DDM parameters. Based on above summarized findings, we hypothesized that aging would be associated with increases in RT, SAT, boundary separation, and non-decision time as well as with reductions in NAA, GABA, Glx, FA and FR and increases in choline, myoinositol, glutamate, MD, RD and AD in all gray matter ROIs and white matter pathways. We further hypothesized, that age-related metabolic and microstructural differences in both bottom-up sensory processing areas (OCC, optic radiation, ILF) and top-down motor execution areas (SLF, fornix, ACC, PPC) would account for differences in overall RTs, and in non-decision time, as the latter reflects both sensory and motor execution processes. In contrast, only regions involved in top-down decision-making (SLF, fornix, ACC, PPC) were expected to predict differences in SAT and boundary separation. No specific hypotheses regarding drift rate were generated given the ambiguity of findings in the literature.

## Methods

### Participants

Participants were recruited from the School of Psychology community participant panel at Cardiff University and consisted of younger (aged 18–29) and older (aged 62–80) adults. Twenty-five participants were recruited into each group, all of whom provided informed written consent prior to taking part in the study in accordance with the Declaration of Helsinki (Cardiff University School of Psychology Ethics committee reference 18.06.12.5313). All participants were cognitively healthy, i.e., had a Montreal Cognitive Assessment (MOCA) score ≥ 26. Participants also completed MRI screening prior to the study, excluding any participants with MRI contraindications such as metallic or electronic bodily implants, some dental work and some tattoos, subject to radiographer assessment. Individuals with visual impairments, such as visual field loss or glaucoma were also excluded from the study. Table 1 summarises participants’ demographic information as well as their mean performance on cognitive and visual screening tasks. Both groups were comparable with regards to sex, handedness, years of education, and visual acuity. All participants had normal or corrected normal visual acuity with Snellen Fractions ≥ 1. The older group performed slightly better on the Test of Premorbid Functioning UK version (TOPF-UK) [99], which involves reading out a list of irregular words, and provides an estimate of verbal intelligence.

**Table 1:** Demographic and baseline cognitive scores for younger and older adults. Mean and standard deviation (SD) for younger and older adults’ performance. MOCA = MOntreal Cognitive Assessment, TOPF-UK = Test Of Premorbid Functioning, UK-edition.

	**Younger Mean (SD)**	**Older Mean (SD)**	**t_(49)_-value** **(p-value)**
Age	21.56 (2.76)	68.36 (6.1)	34.8 (<0.001)
Sex	Male (8) Female (17)	Male (12) Female (13)	–
Handedness	Left (1) Right (24)	Left (4) Right (21)	–
Years of education	16.08 (2.3)	15.64 (4.3)	0.45 (0.66)
MOCA score	28.84 (1.25)	29.04 (1.1)	0.58 (0.56)
Visual acuity (Snellen fraction)	1.91 (0.18)	1.77 (0.3)	1.8 (0.08)
TOPF-UK score	60 (6.43)	64.72 (4.5)	3.0 (0.004)

### Materials & procedure

#### Cognitive and visual testing

Testing was conducted at Cardiff University Brain Research Imaging Centre (CUBRIC), during one visit lasting approximately two hours. Participants completed a visual acuity task and flanker task on a computer which is described in detail below. The task was presented on a 15 inch screen (1440 x 900 native resolution) and responses were recorded using a wireless keypad. The flanker task was written by LR using PsychoPy psychophysics software [63]) for Python (v1.85.6) following the original methodology of the Attention Network Task (ANT) [25] unless otherwise stated. Visual acuity was assessed using the Freiburg Visual Acuity and Contrast Test (FRACT); [3]. Participants viewed the screen from a distance of 2 m (as recommended by test manufacturers) and responded to circular stimuli, where the target was a ‘gap’ in the circle. Stimuli was reduced in size for each correct trial to achieve a Snellen fraction measure of visual acuity.

Reaction times (RTs) were recorded using a modified Attention Network Test (ANT) flanker task [25], and speed accuracy trade-off and DDM parameters were calculated using these RTs. The modified ANT stimuli consisted of five horizontal arrows presented on the screen in which participants were instructed to attend to the central arrow as the target. Central arrows were flanked by horizontal lines (neutral condition), arrows facing in different directions to the target (incongruent condition), or arrows facing in the same direction as the target (congruent condition). During this version of the ANT, stimuli were presented in the same central position on the screen following the presentation of a fixation cross. Participants viewed the screen from a seated position, 400 mm from the computer screen. Participants were instructed to maintain focus on the central fixation point of the screen and respond as quickly and accurately as possible. In accordance with the original study [25], these stimuli subtended 3.08° of visual angle. Fixations were presented for a random variable length of time between 400 and 1600 ms and target stimuli were presented for a maximum of 1700 ms. Participants completed 96 trials (32 trials per condition) in each block, for a duration of 5 blocks. Between blocks, participants were instructed to rest for 30 seconds, before being given a 5 seconds count-down into the following block. The entire task totalled 480 trials and took approximately 12–15 minutes to complete.

#### Speed accuracy trade-off (SAT) calculation

Speed accuracy trade-off (SAT) was calculated from RTs using the linear integrated speed accuracy score (LISAS) [93] method, which combines RT and proportion of error in a linear manner, according to the formula (Equation (1).

(1) $$\mathrm{SAT}=RTj+\frac{\mathrm{SRT}}{\mathrm{SPE}}xPEj$$

where RTj is the mean RT, PEj is the proportion of errors, SRT is the participants’ overall RT standard deviation, and SPE is the participants’ overall standard deviation for the proportion of errors

To assess correlations between RT, SAT, and DDM indices, Spearman’s Rho correlation coefficients were calculated between these measurements. Linear mediation analysis was then used to test for the indirect effects of DDM mediator variables on the direct effects of SAT on mean RT. The significance of indirect and direct effects was assessed with a 95% confidence interval based on bootstrapping with 5000 replacements.

#### Drift diffusion modelling (DDM)

DDM parameters were calculated using the EZ DDM model [96] which was incorporated into an in-house R based custom script. Raw RT and accuracy data for each participant for congruent, neutral and incongruent trial conditions were input into the script in R Studio (v 1.1.463). The script first calculated means and variances of correct RTs. Incorrect trials were not included in the remainder of the analysis (average retained trials = 469). Following this, the script calculated DDM parameters using the equation provided in Wagenmakers et al., [96] under the assumption that trial-to-trial variability was zero and the starting point of each decision process was equidistant from the response boundaries [80]. This resulted in average estimates for non-decision, boundary separation and drift rate for each participant. Details of the mathematical basis for the EZ model can be found in Wagenmakers, Van der Maas & Grasman [96].

#### Magnetic resonance imaging (MR) imaging and spectroscopy

##### MR data acquisition

All MR data were acquired on a Siemens 3 Tesla (T) Magnetom Prisma MR system (Siemens Healthcare GmbH, Erlangen) fitted with a 32-channel receiver head coil at CUBRIC. A 3D, T1-weighted magnetization prepared rapid gradient-echo (MP-RAGE) structural scan was acquired for each participant (TE/TR = 3.06/2250 ms, TI = 850 ms, flip angle = 9 deg, FOV = 256 mm, 1 × 1 × 1 mm resolution, acquisition time = ∼6min). The MPRAGE was used as anatomical reference for the placement of magnetic resonance spectroscopy (MRS) region of interest voxels.

**Fig. 2:**
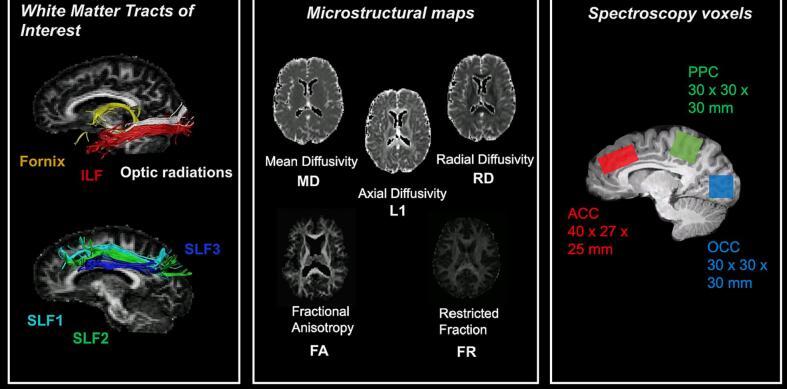
**White matter tracts, microstructural maps and location of spectroscopy voxels for measurements of interest.** ACC = anterior cingulate cortex, ILF = inferior longitudinal fasciculus, PPC = posterior parietal cortex, OCC = occipital cortex, SLF = superior longitudinal fasciculus.

MRS was used to acquire frequency spectra to quantify metabolites of Glx, GABA, NAA, choline, creatine and myoinositol. Single voxel proton spectra were obtained from voxels of interest placed in the occipital cortex (OCC, voxel measuring 30 × 30 × 30 mm^3^), the posterior parietal cortex (PPC, voxel measuring 30 × 30 × 30 mm^3^) and the anterior cingulate cortex (ACC, voxel measuring 27 × 30 × 45 mm). The OCC voxel was placed above the tentorium cerebelli, avoiding scalp tissue in order to prevent lipid contamination to the spectra. The PPC voxel was placed with the posterior edge against the parieto-occipital sulcus, and the ventral edge of the voxel above and parallel to the splenium. Finally, the ACC was placed directly dorsal and parallel to the genu of the corpus callosum. In each voxel, a spectral editing acquisition (MEGA-PRESS, [54]) was performed, involving applying an additional pulse symmetrically about water resonance, providing ‘on’ and ‘off’ editing pulses which allow for the subtraction of peaks which may mask GABA in the spectra (TE/TR = 68/2000 ms, 168 averages, acquisition time = ∼12 min per voxel). Manual shimming was performed before all MRS scans to ensure water-line width of 20 Hz or lower, in order to obtain accurate peaks in the spectra (Fig. 2).

A multi-shell diffusion MRI sequence was also conducted using a high angular resolution diffusion (HARDI) weighted echo-planar imaging (EPI) sequence (TE/TR = 73/ 4100 ms, FOV = 220×220 mm, isotropic voxel size 2 mm^3^, 66 slices, slice thickness 2 mm, acquisition time ∼15 min, 2 × 2 × 2 mm resolution). Five diffusion weightings were applied along gradient directions: b = 200 s/mm^2^ (20 directions), b = 500 s/mm^2^ (20 directions) b = 1200 s/mm^2^ (30 directions), b = 2400 s/mm^2^ (61 directions), b = 4000 s/mm^2^ (61 directions). 12 unweighted (b0) volumes were acquired, interspersed throughout diffusion-weighted scans. In addition, a diffusion reference sequence was acquired for later blip-up blip-down analysis to correct for EPI distortion [104] in which a diffusion weighting of b = 1200 s/mm^2^, and 12 unweighted (b0) images were acquired interspersed throughout the sequence (Fig. 1). Multi-shell diffusion weighted imaging data were acquired to fit the diffusion tensor and the Composite Hindered and Restricted Model of diffusion (CHARMED) [2] to gain microstructural maps of fractional anisotropy (FA), mean diffusivity (MD), radial diffusivity (RD), axial diffusivity (AD), and restricted signal fraction (FR).

##### MR analysis

MRS data were analysed using Totally Automatic Robust Quantification in NMR (TARQUIN) version 4.3.11 [77] in order to determine estimated concentrations of other metabolites of interest (Choline, NAA, Glx, Creatine, Myoinositol). To ensure data quality, metabolites were excluded if the Cramer Rao Lower Bound (CRLB) was above 20 % as recommended [85]. MEGA-PRESS data were analysed using GANNET (GABA-MRS Analysis Tool) version 3.0 [21]. Estimated metabolite values were corrected to account for cerebrospinal fluid (CSF) voxel fraction, and water reference signal was corrected to account for differing water content of CSF, gray matter and white matter. All metabolites were quantified using water as a concentration reference and were expressed as concentration in millimoles per unit (mM) (quality metrics and spectra for MRS raw and fitted data are provided in Supplementary Figure 1).

Multi-shell HARDI data were split by b-value and were corrected for distortions and artifacts using a custom in-house pipeline in MATLAB and Explore DTI [42]. Correction for echo planar imaging distortions was carried out by using interleaved blip-up, blip-down images. Tensor fitting was conducted on the b = 1200 s/mm^2^ data, and the two compartment ‘free water elimination’ (FWE) procedure was applied to improve reconstruction of white matter tracts close to ventricles [62] and to account for partial volume contamination due to cerebrospinal fluid (CSF) which is particularly apparent in older age [53]. Data were fit to the CHARMED model [2] which involved the correction of motion and distortion artefacts with the extrapolation method of Ben-Amitay et al., [6]. The number of distinct fibre populations in each voxel (1, 2 or 3) was determined using a model selection approach [15] and FR maps [2] were then extracted by fitting the CHARMED model to the DWI data, with an in-house script. This resulted in FA, MD, RD, AD and FR maps.

Whole brain tractography was then performed with the dampened Richardson-Lucy (dRL) spherical deconvolution method [18]. Tractography was performed on the b = 2400 s/mm^2^ data to provide better estimation of fibre orientation [95]. The dRL algorithm extracted peaks in the fibre orientation density function (fODF) in each voxel using a step size of 0.5 mm. Streamlines were terminated if directionality of the path changed by more than 45 degrees using standardised in-house processing pipeline at CUBRIC. Manual fibre reconstructions were performed in ExploreDTI v4.8.3 [42]. Tracts of interest were manually drawn on direction encoded colour FA maps in native space. ILF reconstruction was obtained according to protocols by Hodgetts et al. [34] and Wakana et al. [97]. The SLF was subdivided into three subdivisions, the SLF1, 2 and 3 which were delineated according to protocol by Thiebaut de Schotten et al., [89]. The SLF was subdivided based on the distinct contributions of each tract to different functions of attention and executive processing; the SLF 1 being associated with spatial functions and goal-directed attention [89], [61], the SLF2 being associated with orienting attention and integration of dorsal and ventral attention networks [57], and the SLF 3 being associated with reorienting of spatial attention [89], [57]. The fornix was reconstructed by locating the body of the fornix bundle according to Metzler-Baddeley et al. [52], and the optic radiation was delineated by placing a seed region on the white matter of the optic radiation lateral to the lateral geniculate nucleus in the axial plane [90](Fig. 2).

#### Statistical analysis

Statistical analyses were conducted in R-studio (v 1.1.463), SPSS version 27 (IBM) and the PROCESS computational tool for mediation analysis version 4.3 [33]. Data were assessed for normality with the Kolmogorov-Smirnov test and were either analyzed with non-parametric tests or were rank-transformed before conducting parametric testing if they did not fulfill normality. Multiple comparisons were corrected for False Discovery Rate (FDR) to mitigate the likelihood of Type 1 error by employing the Benjamini-Hochberg procedure at 5 % [7]. All reported p-values were two-tailed.

Group differences in rank-transformed DDM parameters, SAT, accuracy, RT, and variance were assessed using independent t-tests. Tractography outcome measures (FA, MD, RD, AD, FR) and metabolite outcome measures (GABA, NAA, Glx, Myoinositol, Choline, Creatine) were compared between older and younger control groups by conducting non-parametric Mann Whitney U tests.

Hierarchical linear regression models were carried out for RT, SAT, and each EZ DDM parameter as dependent variables. Age and TOPF-UK score, the two variables that differed between the groups, were entered into the model first, followed by all metabolic and microstructural measurements in a stepwise fashion. Regression analyses were conducted on rank-ordered variables to account for non-normality of the data.

Following this, Pearson correlations on rank-transformed data were conducted between age, brain predictors and DDM parameters and the directionality of these relationships were explored with mediation analyses [33]. More specifically, linear mediation analysis was used to test for the indirect effect of a mediator variable X (e.g. boundary separation) on the direct effect of variable Y (e.g. RT) on variable Z (e.g. SAT) and *vice versa.* The significance of indirect and direct effect sizes (ES) was assessed with a 95 % confidence interval based on bootstrapping with 5000 replacements [33].

## Results

### Group differences in visual acuity

**Fig. 3:**
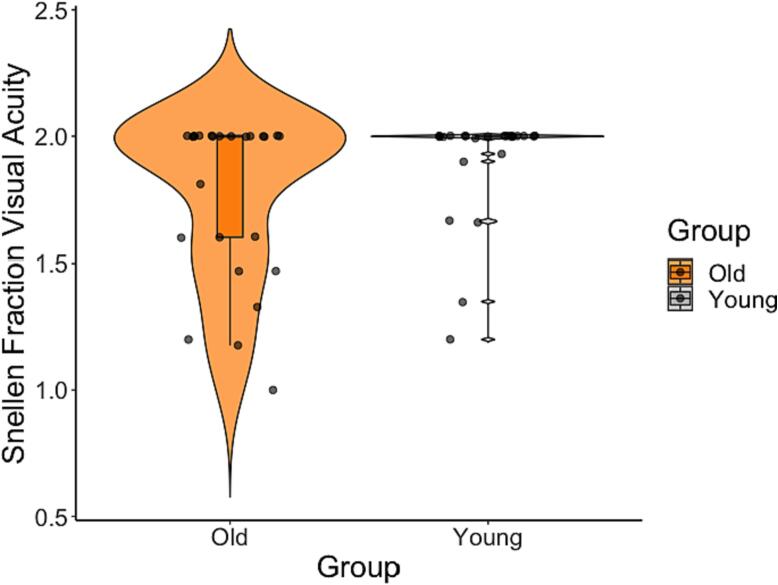
Violin plot with overlaid boxplot for group comparisons between older (orange) and younger (gray) adults’ visual acuity (Snellen Fraction). The boxplot displays the median and the interquartile range and the violin plot the kernel probability density, i.e., the width of the violin area represents the proportion of the data located there. There was no significant difference in visual acuity between younger and older adults. (For interpretation of the references to colour in this figure legend, the reader is referred to the web version of this article.)

No significant differences were found in visual acuity between older and younger age groups (F(1,49) = 1.239, p =.276) (Fig. 3).

### Group differences in RT, SAT, and DDM parameters

**Fig. 4:**
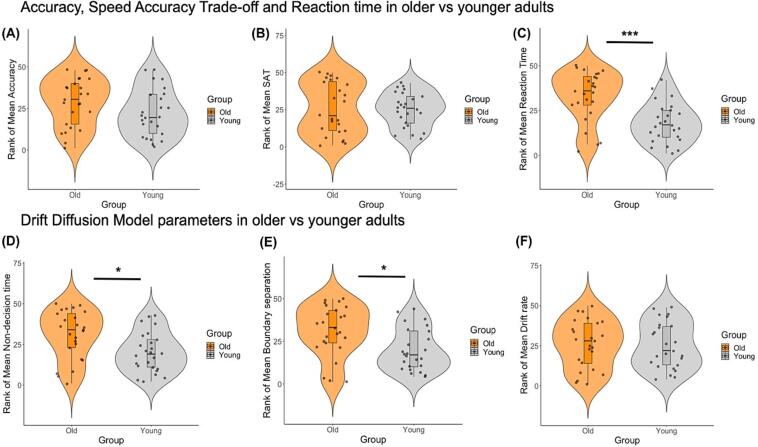
Violin plots with overlaid boxplots for group comparisons between older (orange) and younger (gray) adults’ rank-transformed accuracy, response time (RT), speed accuracy trade-off (SAT) performance and diffusion drift model (DDM) parameters. The boxplots display the median and the interquartile range and the violin plots the kernel probability density, i.e., the width of the violin area represents the proportion of the data located there. Older participants showed increased RT (mean rank-transformed RT_old_ = 33, SD = 14.1; mean rank-transformed RT_young_ = 18, SD = 10.9), boundary separation values (mean rank-transformed boundary separation_old_ = 31, SD = 14.8; mean rank-transformed boundary separation_young_ = 20, SD = 12.3) and non-decision time (perceptual and motor processing) (mean rank-transformed non-decision time_old_ = 30.9, SD = 15; mean rank-transformed non-decision time_young_ = 20.1, SD = 12.2). *** p_FDRcor_ < 0.001, * p_FDRcor_ < 0.05. (For interpretation of the references to colour in this figure legend, the reader is referred to the web version of this article.)

Independent t-tests on rank-transformed data revealed that older compared to younger adults showed larger RT (t(48) = 4.2, p_FDRcor_ = 0.0007) (Fig. 4C), non-decision time (t(48) = 2.9, p_FDRcor_ = 0.016) (Fig. 4D) and boundary separation values (t(48) = 2.9, p_FDRcor_ = 0.016) (Fig. 4E).

No group differences were observed for accuracy (t(48) = 1.6, p = 0.12) (Fig. 4A), SAT (t(48) = 0.18, p = 0.87) (Fig. 4B) or drift rate (t(48) = 0.16, p = 0.87) (Fig. 4F).

### MRI results

#### Metabolic differences between older and younger adults

**Fig. 5:**
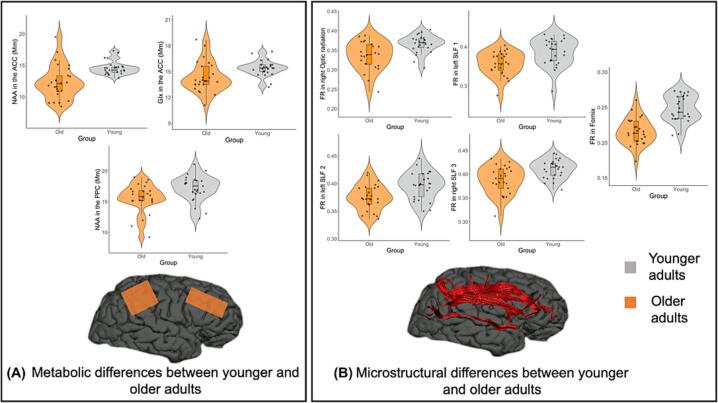
Metabolic and microstructural differences between younger and older adults. (A) Significant group comparisons for metabolites in voxels of interest between older and younger adults (B) Significant group comparisons for FR in tracts of interest between older and younger adults. FR was significantly lower in the fornix, optic radiation, SLF1, 2 and 3 in older adults (orange) in comparison to younger (gray) adults. **p <.001, *p <.0.05. (For interpretation of the references to colour in this figure legend, the reader is referred to the web version of this article.)

Group comparisons between older and younger participants showed no significant differences in GABA levels in the ACC, OCC or the PPC. Older participants had significantly lower Glx (U = 189, p =.004) and NAA (U = 109, p <.001) in the ACC than younger adults (Fig. 5a). A trend towards significantly lower myoinositol in older adults in comparison to younger adults in the ACC was also observed (U = 234, p =.058). In the PPC, older adults showed significantly lower NAA (U = 190, p =.011), and a trend towards lower Glx (U = 231, p =.054) than younger adults (Fig. 5a).

#### Diffusion weighted MRI differences between younger and older adults

Significantly lower restricted fraction FR was shown in the older group in the fornix (U = 75, p <.001), right optic radiation (U = 154, p =.001), left SLF1 (U = 152, p =.001), left SLF2 (U = 170, p =.002) and right SLF3 (U = 169, p =.002) (Fig. 5b).

**Fig. 6:**
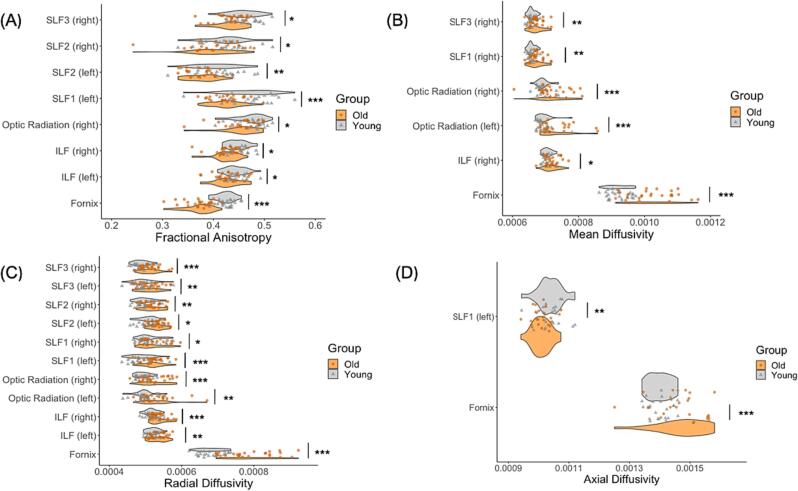
Diffusion tensor imaging (DTI) differences between younger and older adults. Significant group comparisons (p <.0.05) for tract fractional anisotropy (A), mean diffusivity (B), radial diffusivity (C) and axial diffusivity (D) between older and younger adults. * p_FWEcor_ < 0.05, ** p_FWEcor_ < 0.01, p_FWEcor_ < 0.001.

Significantly higher FA in the fornix (U = 22, p <.001), right optic radiation (U = 207, p =.027), left ILF (U = 203, p =.014), right ILF (U = 157, p =.001), left SLF1 (U = 131.5, p <.001), left SLF2 (U = 195, p =.009), and right SLF2 (U = 218, p =.029), and right SLF3 (U = 163, p =.001) was found in younger adults in comparison to older adults. Significantly higher MD in the older group in comparison to the younger group was found in the fornix (U = 84, p <.001), left optic radiation (U = 78, p <.001), right optic radiation (U = 131, p <.001), right ILF (U = 203, p =.014), right SLF1 (U = 175, p =.003), right SLF3 (U = 175, p =.003). Radial diffusivity was significantly higher in the older relative to the younger group in all but two (right SLF1, left SLF2) tracts of interest: fornix (U = 39, p <.001), left optic radiation (U = 145, p =.001), right optic radiation (U = 139, p <.001), left ILF (U = 189, p =.007), right ILF (U = 143, p <.001), left SLF1 (U = 130, p <.001), right SLF2 (U = 178, p =.003), left SLF3 (U = 197.5, p =.010), right SLF3 (U = 135.5, <0.001) (Fig. 6). Significantly greater axial diffusivity in older adults was found in the fornix (U = 529, p < 0.001), and significantly lower axial diffusivity in older adults was found in the SLF1 left (U = 178, p = 0.005).

### Correlations between response latency, SAT, and DDM parameters

**Fig. 7:**
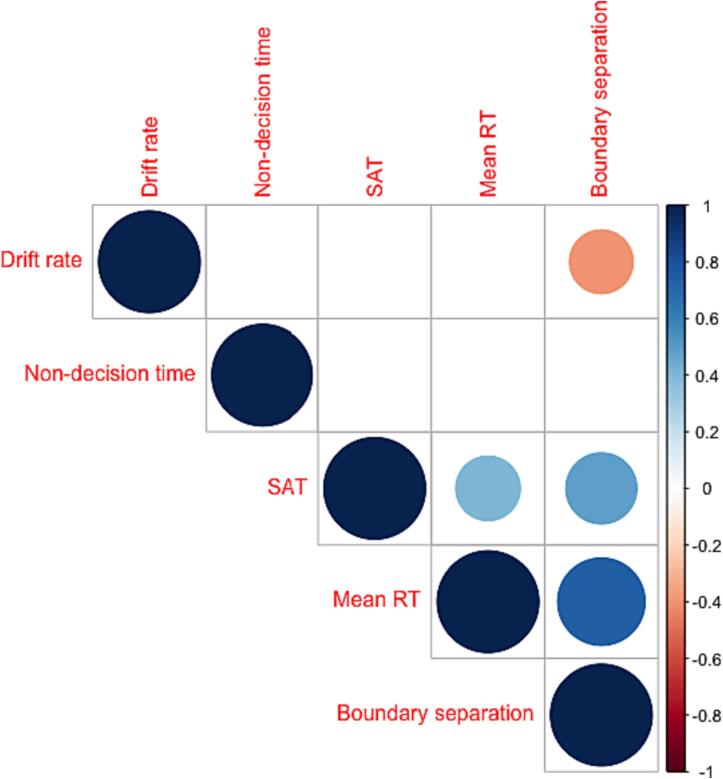
Correlation matrix between Speed Accuracy Trade-off (SAT), mean Reaction Time (RT), and diffusion drift model (DDM) parameters**.** Mean RT and boundary separation (p <.001), SAT and boundary separation (p <.001) and mean RT and SAT (p <.05) were positively correlated (blue shades). Drift rate and boundary separation were negatively correlated (p <.05) (red shade). (For interpretation of the references to colour in this figure legend, the reader is referred to the web version of this article.)

Significant positive correlations were observed between mean RT and boundary separation (Rho = 0.74, p_FWEcor_ < 0.00000001), between SAT and boundary separation (Rho = 0.49, p_FWEcor_ = 0.002), and between mean RT and SAT (Rho = 0.4, p_FWEcor_ = 0.013). Drift rate and boundary separation were negatively correlated (Rho = -0.4, p_FWEcor_ = 0.013) (Fig. 7). Non-decision time did not correlate with any of the other cognitive variables (Fig. 7).

**Fig. 8:**
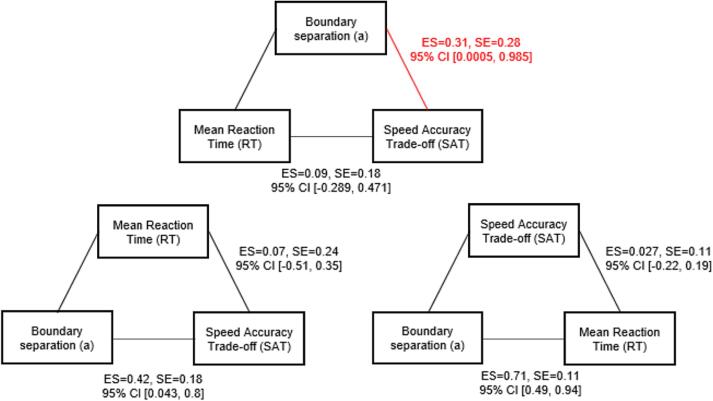
Mediation analyses between Speed Accuracy Trade-off (SAT), boundary separation and mean Reaction Time (RT). Boundary separation had a significant indirect effect on SAT, removing the direct effect of mean RT on SAT. No other direct or indirect effects were significant. ES = Effect size.

Mediation analysis revealed that boundary separation had a significant indirect effect (indirect ES of boundary separation = 0.31, SE = 0.28, 95 % CI 0.0005–0.985) and removed the direct effect of mean RT on SAT (remaining ES of mean RT on SAT = 0.09, SE = 0.18, 95 % CI −0.289 – 0.471) (Fig. 8). In contrast the inclusion of mean RT or SAT as mediator variables did not have any indirect effects on the direct effect of boundary separation on SAT (indirect ES of mean RT = 0.07, SE = 0.24, 95 % CI −0.51–0.35; direct ES of mean boundary separation on SAT = 0.42, SE = 0.18, 95 % CI 0.043 – 0.8) or on mean RT (indirect ES of SAT = 0.027, SE = 0.11, 95 % CI −0.22–0.19; direct ES of mean boundary separation on mean RT = 0.71, SE = 0.11, 95 % CI 0.49 – 0.94). This pattern of results demonstrates that differences in boundary separation accounted for the shared variance in mean RT and SAT.

### Neurobiological predictors of response latencies, SAT, and DDM parameters

Hierarchical linear regression analyses testing for the effects of age, TOPF-UK score, and all microstructural and metabolic brain measurements on mean RT, SAT, and DDM indices were conducted separately for each outcome measure. All models entered age and TOPF-UK score as first predictors followed by the stepwise inclusion of the brain measurements (Supplementary Table 1).

#### Response latencies (RTs) and SAT

Variation in RTs were not accounted for by age and TOPF-UK score alone (adj R2 = 0.01, F(2,37) = 1.2, p = 0.31) but the inclusion of the following microstructural and metabolic brain measurements improved the fit of the model significantly: fornix FA (delta R2 = 0.24, F(1,36) = 12.5, p = 0.001), AD in left optic radiation (delta R2 = 0.14, F(1,35) = 8.9, p = 0.005), RD in right SLF1 (delta R2 = 0.11, F(1,34) = 8.7, p = 0.006), myoinositol in OCC (delta R2 = 0.05, F(1,33) = 4.2, p = 0.048), and AD in right SLF1 (delta R2 = 0.06, F(1,32) = 5.2, p = 0.029). The final model explained 66 % of the variation in RTs (adj R2 = 0.59, F(7,32) = 9.01, p < 0.001) and included the following predictors: fornix FA (beta = -0.9, p_FDRcor_ < 0.0000001), RD in right SLF1 (beta = -0.33, p_FDRcor_ = 0.019), myoinositol in OCC (beta = 0.38, p_FDRcor_ = 0.019), TOPF-UK score (beta = 0.32, p_FDRcor_ = 0.025), AD in right SLF1 (beta = 0.35, p_FDRcor_ = 0.034) and AD in left optic radiation (beta = -0.26, p_FDRcor_ = 0.034).

**Fig. 9:**
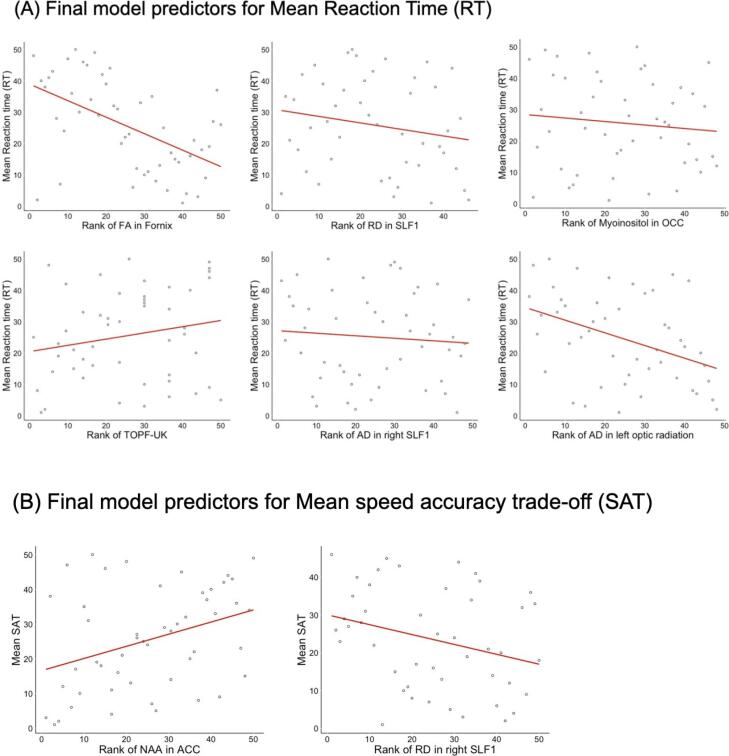
Slopes of the regression lines for the brain predictors in the final model for rank of mean reaction time (RT) (A) and mean Speed Accuracy Trade-off (SAT) (B). Significant predictors (p <.05) of rank mean RT and rank mean SAT included in final hierarchical models.

Age and TOPF-UK score alone did not predict variability in SAT (adj R2 = -0.025, F(2,37) = 0.53, p = 0.6). The inclusion of the following metabolic and microstructural measurements improved the fit of the model significantly (adj R2 = 0.42, F(5,34) = 4.9, p = 0.002): NAA in the ACC (delta R2 = 0.21, F(1,36) = 10.12, p = 0.003), RD in right SLF1 (delta R2 = 0. 1, F(1,35) = 5.3, p = 0.03) and FA in right SLF1 (delta R2 = 0.08, F(1,34) = 4.5, p = 0.04). In the final model SAT was significantly predicted by NAA in the ACC (beta = 0.5, p_FDRcor_ = 0.015) and RD in the right SLF1 (beta = -0.9, p_FDRcor_ = 0.015) (Fig. 9).

#### DDM parameters

Variation in boundary separation was not explained by age and TOPF-UK score (adj R2 = 0.029, F(2,37) = 1.6, p = 0.22) but the inclusion of fornix FA (delta R2 = 0.20, F(1,36) = 10.12, p = 0.003) and FR in the right ILF (delta R2 = 0.08, F(1,35) = 4.6, p = 0.04) improved the fit of the model significantly (adj R2 = 0.29, F(4,35) = 5.01, p = 0.003). In the final model fornix FA (beta = -0.8, p_FDRcor_ = 0.002) was the only significant predictor for variation in boundary separation.

Similarly, variation in non-decision time was not explained by age and TOPF-UK score alone (adj R2 = -0.02, F(2,37), p = 0.5). The inclusion of the following metabolic and microstructural measurements improved the model fit significantly (adj R2 = 0.47, F(6,33) = 6.7, p < 0.001): NAA in the ACC (delta R2 = 0.23, F(1,36) = 11.5, p = 0.002), AD in the right ILF (delta R2 = 0.15, F(1,35) = 8.9, p = 0.005), creatine in the OCC (delta R2 = 0.07, F(1,34) = 4.3, p = 0.045) and GLx in PPC (delta R2 = 0.07, F(1,33) = 4.9, p = 0.034). In the final model NAA in the ACC (beta = -0.45, p_FDRcor_ = 0.015), AD in the right ILF (beta = 0.38, p_FDRcor_ = 0.015), and creatine in the OCC (beta = 0.32, p_FDRcor_ = 0.03) predicted non-decision time significantly.

**Fig. 10:**
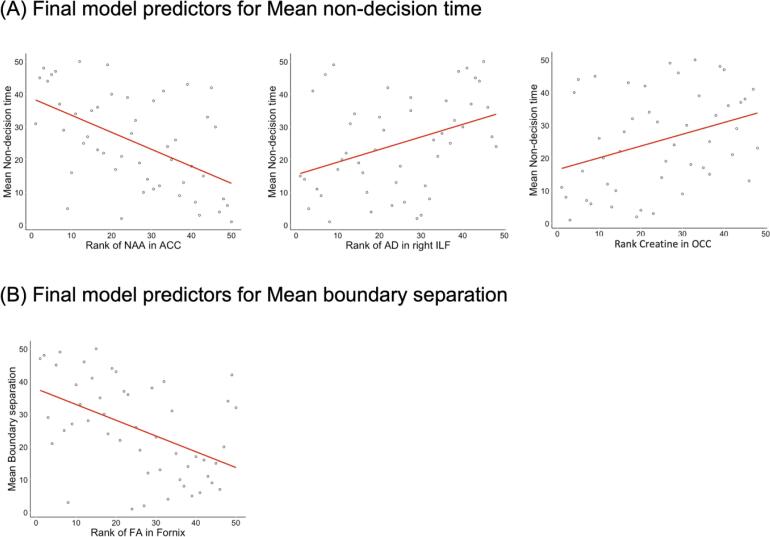
Slopes of the regression lines for the brain predictors in the final model for rank of mean non-decision time (A) and mean boundary separation (B). Significant predictors (p <.05) of rank mean non-decision time and rank mean boundary separation included in final hierarchical models.

Finally, age and TOPF-UK score did not account for variation in drift rate (adj R2 = 0.01, F(2,37), p = 0.32) and the inclusion of brain measurements did not improve the model fit significantly (adj R2 = 0.12, F(3,36) = 2.6, p = 0.07) (Fig. 10).

#### Regression analyses controlling for age and TOPF score separately

As the variables age and TOPF-UK were positively correlated (rho = 0.4, p = 0.003) we explored any biases in the regression modelling by repeating the analyses controlling for age and TOPF-UK separately. Accounting for TOPF-UK but not age led to the same pattern of results for all cognitive variables as described above. Accounting for age but not TOPF-UK led to the same results for non-decision time and SAT but changed the following results: The final model for boundary separation (adj R2 = 0.42, F(5,34) = 6.07, p < 0.001) included predictors of fornix FA (beta = -0.8, p_FDRcor_ = 0.0001), choline in PPC (beta = -0.36, p_FDRcor_ = 0.02), RD in right SLF1 (beta = -0.3, p_FDRcor_ = 0.04) and myoinositol in OCC (beta = -0.3, p_FDRcor_ = 0.04). Drift rate variation was significantly explained by a model (adj R^2^ = 0.12, F(2,37) = 3.7, p = 0.035) with predictors of age (beta = 0.4, p_FDRcor_ = 0.03) and FA in right ILF (beta = 0.4, p_FDRcor_ = 0.03). Finally, variation in RT (adj R2 = 0.49, F(4,35) = 10.2, p < 0.001) were accounted for by fornix FA (beta = -0.63, p_FDRcor_ = 0.002), AD in left optic radiation (beta = -0.37, p_FDRcor_ = 0.01) and RD in right SLF1 (beta = -0.35, p_FDRcor_ = 0.01). Age alone did not predict any of the cognitive variables.

#### Correlation analyses between age, fornix FA, and RT

Spearman correlation coefficients were calculated between age, fornix FA, RT and boundary separation to explore the directionality of these relationships. Fornix FA correlated negatively with age (Rho = -0.75, p_FWEcor_ < 0.00000001), boundary separation (Rho = -0.48, p_FWEcor_ = 0.007), and RT (Rho = -0.53, p_FWEcor_ = 0.002). A significant positive correlation was present between age and RT (Rho = 0.35, p_FWEcor_ = 0.016) and a trend for a positive correlation between age and boundary separation (Rho = 0.26, p = 0.06).

**Fig. 11:**
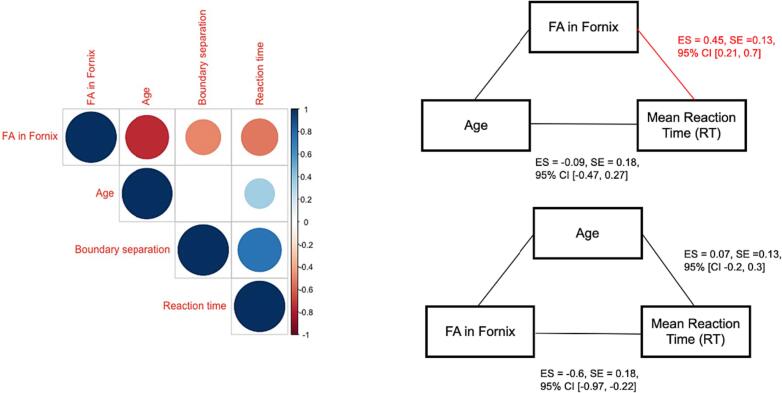
Correlation matrix and mediation analyses investigating the relationship between age, fractional anisotropy (FA) in the fornix, mean reaction time (RT) and boundary separation value. Correlation matrix (left) shows significant negative correlations (red shade) (p <.05) between fornix FA, age, mean RT and boundary separation. A significant positive correlation (blue shade) was present between age and RT. Mediation analyses (right) shows a significant indirect effect of fornix FA on mean RT, which removed the direct effect of age on mean RT. Age as an indirect predictor did not remove the effect of fornix FA on mean RT. (For interpretation of the references to colour in this figure legend, the reader is referred to the web version of this article.)

Mediation analysis demonstrated that fornix FA had a significant indirect effect (ES = 0.45, SE = 0.13, 95 % CI 0.21–0.7) and removed the direct effect of age on mean RT (remaining ES = -0.09, SE = 0.18, 95 % CI −0.47 – 0.27). In contrast the inclusion of age as mediator variable did not have an indirect effect (ES = 0.07, SE = 0.13, 95 % CI −0.2–0.3) on the direct effect of fornix FA on RT (ES = -0.6, SE = 0.18, 95 % CI −0.97 – −0.22). This pattern of results suggests that age-related response slowing is mediated by the age-related decline in fornix microstructure but not *vice versa* (Fig. 11).

## Discussion

The aim of this study was to investigate the cognitive and neurobiological substrates of age-related response slowing in visuo-perceptual decision making. For this purpose, we modelled older and younger adults’ RT data from the ANT flanker task with the EZ DDM to derive three cognitive components thought to contribute to response slowing in aging. These were the non-decision time, that reflects the time needed for bottom-up sensory and top-down motor execution processes, the boundary separation value, that reflects the degree of conservatism regarding a response decision criterion, and the drift-rate, the speed with which information was accumulated to reach a response threshold. Furthermore, we employed advanced multi-shell diffusion-weighted MRI and MRS techniques to acquire estimates of microstructural and metabolic properties of gray matter ROIs and white matter connections in visual-perceptual and attention networks. We then tested which of these brain measurements were predictive of differences in cognitive parameters for the purpose of elucidating the neurobiological basis of any age-related differences in visuo-perceptual decision making.

### Age effects on response times, SAT, and DDM parameters

Consistent with the literature [72], [68], [88] we found age-related increases in RTs, boundary separation, and non-decision time. These differences were observed in participants without visual acuity impairments as measured with the Snellen Fraction, thus were not due to reduced eyesight in older participants. Contrary to our hypotheses, older adults did not show increased SAT, as measured with LISAS, and did not differ in accuracy or diffusion drift rates from younger adults.

Slowed response times while maintaining accuracy is a characteristic pattern observed in aging and is generally thought to reflect a shift to a more conservative decision criterion [68], [79], [83], [86]. Consistent with this view mediation analysis revealed that differences in boundary separation accounted for the shared variance between SAT and RTs but not *vice versa*, suggesting that the shift to a more conservative response strategy was the main contributor to response slowing in aging. Drift rate was negatively correlated with boundary separation as the more conservative the response strategy the longer it took to reach a response threshold criterion. While age-related increases in non-decision time were observed and accord with accounts of low-level sensory degradation and slowing of motor execution, these processes did not contribute to overall response slowing or to the increases in boundary separation.

### Age effects on microstructural and metabolic brain measurements

The main aim of our study was to investigate the neurobiological basis of differences in cognitive components thought to underpin age-related response slowing. For this purpose, we acquired microstructural and metabolic measurements with advanced diffusion-weighted MRI and MRS from key regions within visual perceptual and attention-executive networks. Regions involved in bottom-up visuo-sensory and perceptual processing comprised the OCC as well as the optic radiation and ILF while areas hypothesized to mediate top-down decision-making processes were the ACC, PPC, SLF and fornix.

In accord with the literature [55], [78], [100], we observed age-related reductions in FA and increases in MD, RD, and AD in all pathways, providing further evidence of the detrimental effects of age on white matter microstructure. The nature of these age-related white matter differences is difficult to infer from DTI indices alone because they are sensitive to biological (e.g. myelin, axon density) and geometrical properties (e.g. crossing/kissing fibers) of fibers [15]. The restricted signal fraction FR from CHARMED provides a complementary index that can be interpreted as an estimate of axon density [2]. Age-related reductions of FR were present in the optic radiation, fornix, and SLF fibers, suggesting a decrease in the density of axons due to a loss of myelin and/or axons secondary to Wallerian degeneration in aging [15].

With regards to brain metabolites and consistent with previous studies ([46]; see for review [32]), older relative to younger adults showed reduced levels of NAA and Glx in the ACC and lower levels of NAA in the PPC. NAA and Glx are both markers of neuronal metabolism [58] and are considered to play a key role in energy metabolism in neural mitochondria [43]. These findings are consistent with accumulating evidence of energy depletion as a key component of biological aging [74]. With normal aging, the accumulation of biological ‘imperfections’ such as protein aggregation are thought to impair mitochondrial function and cause low-level inflammation, resulting in reduced glucose uptake, synaptic deterioration, and gliosis, and in turn to further energy reduction [13]. The pattern of our results suggest that aging affects neuronal metabolism in frontal and parietal attention regions more than in the OCC, consistent with evidence suggesting that aging is particularly associated with a reduction in mitochondrial energy metabolism in frontal brain regions [75], [76], [101].

### Neurobiological basis of age-related differences in cognitive variables

To study the neurobiological underpinnings of age-related response slowing, hierarchical regression analyses testing for the effects of microstructural and metabolic brain measurements on RT, SAT, and DDM parameters while controlling for age and TOPF-UK (together and separately) were conducted.

The observed patterns of brain-cognition relationships were broadly consistent with our hypotheses. Differences in overall RTs were predicted by microstructural and metabolic estimates from a network of top-down (fornix FA, right SLF1 AD and RD) and bottom-up brain regions (OCC myoinositol, left optic radiation AD) together with verbal IQ (TOPF-UK), reflecting that overall response latencies were a function of all processes involved in visuo-perceptual decision-making. Furthermore, we observed that differences in brain indices in top-down regions involved in decision making predicted boundary separation (fornix FA, PPC choline, right SLF1 RD) and SAT (ACC NAA, right SLF1) while a combination of measurements from bottom-up sensory (OCC creatine, right ILF AD) and top-down motor execution (ACC NAA) regions predicted non-decision time that captured low level sensory function and motor execution. Due to the ambiguities in the literature, we did not generate a specific hypothesis for drift rate but found that age and FA in the right ILF predicted drift rate when TOPF-IQ was removed from the analysis. Importantly, age alone did not predict any of the cognitive variables, including those (RT, boundary separation, non-decision time) that were found significantly increased in older relative to younger adults. This suggests that age-related response slowing and increases in DDM parameters were not driven by age *per se* but by the neurobiological changes that accompany aging.

This interpretation is further substantiated by the finding that fornix FA was the strongest predictor of overall RT (beta = -0.9, p_FDRcor_ < 0.00001) and boundary separation (beta = -0.8, p_FDRcor_ = 0.002), demonstrating that lower fornix FA values were associated with slower response times (Rho = -0.5) and higher boundary separation values (Rho = -0.48). In addition, fornix FA was highly inversely correlated with age (Rho = -0.75), in accord with well-established findings of age-related decline in fornix microstructure (e.g. [20], [52], [53], [84]). Additional mediation analyses revealed that differences in fornix FA removed the effects of age on RT but not *vice versa,* i.e., the inclusion of age did not remove the effects of fornix FA on RT. Thus, age-related response slowing was mediated by the age-related decline in fornix microstructure.

The fornix is the main white matter pathway that connects the hippocampal formation with cortical and subcortical sites beyond the temporal lobe [65]. The precommissural branch of the fornix principally innervates brain regions known to be important for executive function and decision-making notably the prefrontal cortex, ACC, and the basal forebrain, while postcommissural fibers innervate anterior thalamus and mamillary bodies [65]. The role of the hippocampus and the fornix in contextual learning [92] and episodic memory [1], [91] and in the age-related decline of these functions is well-established [20], [52]. In addition, evidence from neuroimaging and lesion studies of the involvement of the hippocampus and the fornix in complex visual discrimination tasks [41], [67] suggests that medial temporal lobe structures are not only involved in the processing of mnemonic but also visuo-perceptual representations [30], [41]. Relevant to decision-making performance, it has been proposed that hippocampal-prefrontal cortex interactions allow contextual cues to override predominant conditioned responses if the context is inconsistent (for instance in the discongruent flanker condition) to aid the selection of previously learned context-appropriate responses (for example the selection of responses congruent with the direction indicated by the target but not by the flanker arrows) [92]. Precommissural fornix fibers that connect the hippocampus with the prefrontal cortex allow efficient interaction between these regions.

In our study, fornix FA was the largest predictor for boundary separation together with choline in PPC, RD in right SLF1 and myoinositol in OCC (when TOPF score was removed from the analysis). Following Turner and Becker’s contextual cuing account (2008), we propose that the hippocampus *via* the fornix provides prefrontal and parietal structures of the right-lateralized executive network [89] with contextual information to facilitate appropriate response selection. Thus, the fornix may play an important role in the accumulation of contextual information required by executive regions to reach a response decision threshold criterion. If fornix microstructure is compromised by aging, this process will become noisier, and more information needs to be accumulated before a decision can be reached. In this way, the fornix as part of an extended hippocampal-prefrontal decision-making network may contribute to the adoption of a more conservative response strategy that underpins age-related slowing in visual discrimination.

We also observed an increase of non-decision time with age, which was predicted by a reduction of NAA in the ACC and increases of creatine in the OCC and AD in the right ILF. This suggest that neuronal decline in visual sensory-perceptual networks and regions involved in motor action control (ACC), due to reduced energy metabolism and neural activity, myelin damage and/or axonal loss, contribute to the loss of sensory and motor functions in older age. However, inconsistent with the view that age-related sensory degradation and/or increases in motor noise, contribute to response slowing and the adoption of a more conservative response strategy, differences in non-decision time were unrelated to differences in boundary separation and overall RT. Finally, drift–diffusion was predicted by age and right ILF FA, suggesting that the speed with which visual information is accumulated depends on the integrity of association fibers, that connect the occipital with temporal cortices.

## Limitations of the study

The sample size of 25 participants per age group in this study was relatively small and it would be advantageous to replicate the here observed findings in a larger cohort. However, it is noteworthy that our study successfully replicated well-established age effects, including the increase of overall RTs, non-decision time, and boundary separation, and the widespread decline of white matter microstructure, notably in the fornix, and of ACC and Glx in older age. Our main findings were based on moderate to large effect sizes, suggesting that despite the modest sample size, the study was appropriately powered to identify some cognitive and neurobiological correlates of age-related slowing in visuo-perception.

The present study adopted the two-choice Eriksen flanker task as a well-established visuo-perceptual response conflict paradigm that allows the modelling of RTs with DDM and has been shown to rely on prefrontal cortex and striatal decision-making regions [87]. The findings and inferences reported here are based on this experimental paradigm and may not transfer to human decision-making behaviours in other paradigms, such as lexical or gambling-related decision-making.

Our results suggest that under the specific task conditions employed here boundary separation may provide a more sensitive measurement of SAT than LISAS for which no age effect was present. Our instructions for the flanker task did not seek to experimentally manipulate accuracy-speed trade-offs by emphasising speed over accuracy or *vice versa*. It could therefore be that opposing between-subject trade-offs may have masked any SAT differences between the age groups. In other words, the increased speed by one participant may have been compensated by the increased accuracy by another. LISAS is a linear measure of SAT that has been shown to be insensitive to SAT, if the SAT effects are linearly balanced across participants [93]. Thus, LISAS may have been a suboptimal choice to estimate SAT in the present study.

Finally, the neurobiological interpretation of the differences in white matter microstructural indices needs to be done with caution. While we observed age-related reductions in FR from CHARMED in the fornix, optic radiation and SLF, suggesting a decline in axon density and/or axonal myelination in these pathways, the FR index did not significantly predict any cognitive components. In contrast, the DTI measurements of FA, AD and RD were identified as significant brain predictors of cognition in the regression analyses. This pattern of results suggests that DTI indices that capture a variety of age-related differences in white matter microstructure due to differences in biological properties, such as loss of myelin and axons and increased inflammation, and differences in the geometry and complexity of fibers are more sensitive brain predictors of cognitive change than the FR index that is thought to be more sensitive to specific biological properties of white matter (axon density). DTI and FR measures were corrected for partial volume effects with the Free Water Elimination Method [62], but this method cannot completely rule out that atrophy-induced free water contamination may have biased these indices. This may have particularly affected the DTI indices in regions susceptible to partial volume contamination notably the fornix [53], [60] and may have made these indices more sensitive to age-related tissue atrophy.

## Conclusions

This study provides new insights into the cognitive and neurobiological underpinnings of age-related response slowing. Our findings are consistent with the view that age-related slowing in visual discrimination is primarily driven by the adoption of a more conservative response strategy. They suggest that this response shift was driven by the effects of aging on neural structures that contribute to decision-making processes rather than by age *per se*. The fornix as the principal connection between the hippocampal formation and anterior decision-making structures was identified as a key predictor of age-related response slowing and the adoption of a conservative response strategy. We propose that age-related fornix decline may result in noisier communication of contextual information to anterior decision-making regions and hence to a more conservative response strategy.

## CRediT authorship contribution statement

**LaurenRevie:** Conceptualization, Data acquisition, Formal analysis, Writing – original draft, Preparation of Figures and Tables. **Claudia Metzler-Baddeley:** Conceptualization, Formal analysis, Funding acquisition, Supervision, Writing – original draft, Writing – review & editing.

## Declaration of competing interest

The authors declare that they have no known competing financial interests or personal relationships that could have appeared to influence the work reported in this paper.
