# Supplementary material for: Age-related fornix decline predicts conservative response strategy-based slowing in perceptual decision-making
Source: Aging Brain. 2024 Jan 24;5:100106. doi: 10.1016/j.nbas.2024.100106 (PMC10838937; doi:10.1016/j.nbas.2024.100106)
Supplement: Supplementary data 1 [file mmc1.docx]

Supplementary Table 1: Summary of the results of the hierarchical regression models for RT, SAT and DDM parameters, entering age and TOPF as first model followed by the stepwise regression of all brain measurements

| **Speed accuracy trade-off (SAT)** | | | | | |
| --- | --- | --- | --- | --- | --- |
| **Predictors** | **R^2^** | **Adj R^2^** | **R^2^ change** | **F change** | **Model**  **significance** |
| Age  TOPF | 0.028 | -0.03 | 0.028 | 0.208 | F_(2,37)_ = 0.53  p = 0.595 |
| Age  TOPF  NAA in ACC | 0.24 | 0.18 | 0.213 | 10.12 | F_(3,36)_ = 3.81  p = 0.018 |
| Age  TOPF  NAA in ACC  RD in right SLF1 | 0.34 | 0.27 | 0.1 | 5.33 | F_(4,35_) = 4.53  p = 0.005 |
| Age  TOPF  NAA in ACC  RD in right SLF1  FA in right SLF1 | 0.42 | 0.33 | 0.08 | 4.52 | F(_5,34)_ = 4.89  p = 0.002 |
| **Mean Reaction time (RT)** | | | | | |
| **Predictors** | **R^2^** | **Adj R^2^** | **R^2^ change** | **F change** | **Model**  **significance** |
| Age  TOPF | 0.06 | 0.01 | 0.06 | 1.2 | F_(2,37)_ = 1.2  p = 0.312 |
| Age  TOPF  Fornix FA | 0.303 | 0.245 | 0.24 | 12.48 | F_(3,36_) = 5.2  p = 0.004 |
| Age  TOPF  Fornix FA  AD in left optic radiation | 0.445 | 0.382 | 0.14 | 8.98 | F(_4,35)_ = 7.1  p < 0.001 |
| Age  TOPF  Fornix FA  AD in left optic radiation  RD in right SLF1 | 0.558 | 0.493 | 0.113 | 8.67 | F_(5,34)_ = 8.6  p < 0.001 |
| Age  TOPF  Fornix FA  AD in left optic radiation  RD in right SLF1  Myoinositol in OCC | 0.608 | 0.537 | 0.05 | 4.237 | F_(6,33)_ = 8.5  p < 0.001 |
| Age  TOPF  Fornix FA  AD in left optic radiation  RD in right SLF1  Myoinositol in OCC  AD in right SLF1 | 0.663 | 0.590 | 0.055 | 5.216 | F_(2,37)_ = 9.01  p < 0.001 |
| **Mean Non-decision time (t)** | | | | | |
| **Predictors** | **R^2^** | **Adj R^2^** | **R^2^ change** | **F change** | **Model**  **significance** |
| Age  TOPF | 0.036 | -0.016 | 0.036 | 0.688 | F_(2,37)_ = 0.68  p = 0.509 |
| Age  TOPF  NAA in ACC | 0.27 | 0.21 | 0.233 | 11.45 | F_(3,36_) = 4.4  p = 0.01 |
| Age  TOPF  NAA in ACC  AD in right ILF | 0.42 | 0.35 | 0.15 | 8.9 | F(_4,35)_ = 6.26  p < 0.001 |
| Age  TOPF  NAA in ACC  AD in right ILF  creatine in OCC | 0.48 | 0.41 | 0.066 | 4.32 | F_(5,34)_ = 6.35  p < 0.001 |
| Age  TOPF  NAA in ACC  AD in right ILF  creatine in OCC  Glx in PPC | 0.55 | 0.47 | 0.067 | 4.9 | F_(6,33)_ = 6.7  p < 0.001 |
| **Mean Boundary separation (a)** | | | | | |
| **Predictors** | **R^2^** | **Adj R^2^** | **R^2^ change** | **F change** | **Model**  **significance** |
| Age  TOPF | 0.079 | 0.029 | 0.079 | 1.591 | F_(2,37)_ = 1.59  p = 0.217 |
| Age  TOPF  Fornix FA | 0.281 | 0.221 | 0.202 | 10.12 | F_(3,36)_ = 4.7  p = 0.007 |
| Age  TOPF  Fornix FA  FR in right ILF | 0.364 | 0.292 | 0.83 | 4.57 | F_(4,35_) = 5.01  p = 0.003 |
| **Mean Drift rate (v)** | | | | | |
| **Predictors** | **R^2^** | **Adj R^2^** | **R^2^ change** | **F change** | **Model**  **significance** |
| Age  TOPF | 0.06 | 0.009 | 0.06 | 1.18 | F_(2,37)_ = 1.18  p = 0.319 |
| Age  TOPF  FA in right ILF | 0.17 | 0.108 | 0.117 | 5.119 | F_(3,36_) = 2.58  p = 0.069 |

Supplementary Table 2: Mean metabolite concentrations in the three cortical regions of interest for the young and old adults.

| **Region of Interest** | **Metabolite** | **Young**  **Mean** | **Young**  **SD** | **Old**  **Mean** | **Old**  **SD** |
| --- | --- | --- | --- | --- | --- |
|  |  | N = 25 | | N = 25 | |
| Occipital cortex | GABA | 4.7016 | 0.4834 | 4.79960 | 0.60611 |
|  | GLx | 13.6760 | 0.87620 | 13.7200 | 2.01453 |
|  | NAA | 17.5108 | 2.41565 | 17.9730^&^ | 2.28685 |
|  | myoinositol | 9.8936 | 1.27030 | 9.1620^&^ | 1.91257 |
|  | choline | 1.8000 | 0.20091 | 1.8374^&^ | 0.28923 |
|  | creatine | 10.8704 | 0.85745 | 10.9175^&^ | 1.45936 |
| Anterior cingulate cortex | GABA | 4.1452 | 0.28397 | 3.9076 | 0.43982 |
|  | GLx | 15.3728 | 0.90590 | 14.3680 | 1.75089 |
|  | NAA | 14.5960 | 1.07127 | 12.3861 | 2.69226 |
|  | myoinositol | 10.3293 | 1.12409 | 9.2002 | 2.35451 |
|  | choline | 2.6010 | 0.25169 | 2.4765 | 0.44072 |
|  | creatine | 10.1418 | 0.63557 | 9.3424 | 1.44127 |
| Posterior parietal cortex | GABA | 4.2300 | 0.37076 | 4.4379^^^ | 0.44991 |
|  | GLx | 14.5000 | 0.96566 | 13.7625^^^ | 1.49516 |
|  | NAA | 17.1692 | 2.07698 | 15.5710^^^ | 2.34532 |
|  | myoinositol | 10.1976 | 1.25723 | 9.1358^^^ | 2.25137 |
|  | choline | 2.1024 | 0.22746 | 2.1545^^^ | 0.37545 |
|  | creatine | 10.6920 | 0.93449 | 10.2680^^^ | 1.57684 |

Abbreviation: GABA = γ- aminobutyric acid, GLx = glutamate/glutamine, NAA = N-acetyl aspartate, SD = standard deviation. ^&^ n = 23, ^^^ n = 24.

Supplementary Figure 1: Quality metrics for MRS raw and fitted data. Metrics from Tarquin fitted data: Full width at half maximum (FWHM), Signal to noise ratio (SNR), Ratio of fit residual to noise (Q), Water FWHM. Metrics from Gannet fitted data: Water FWHM


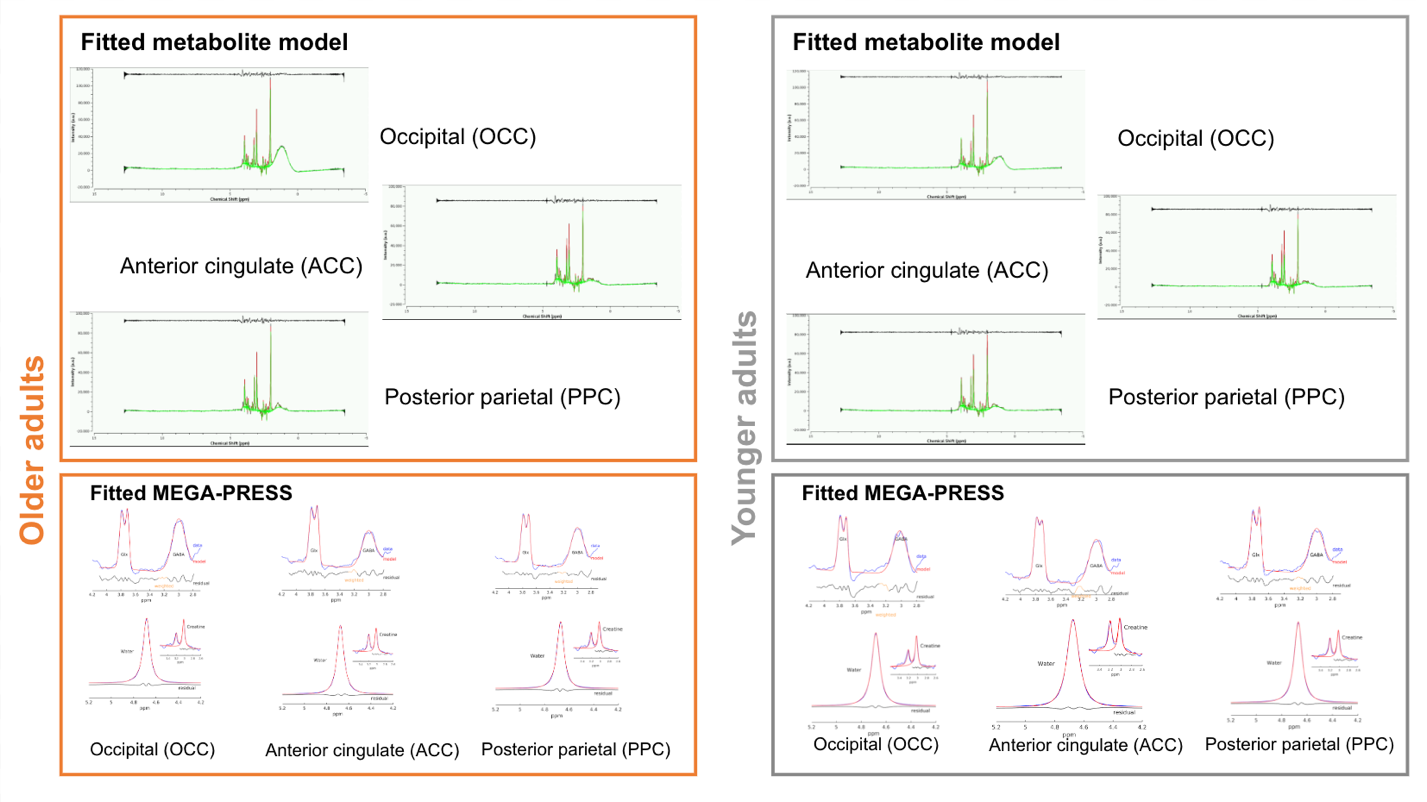


|  | Younger adults | | | Older adults | | |
| --- | --- | --- | --- | --- | --- | --- |
| **Quality metrics** | **OCC** | **ACC** | **PPC** | **OCC** | **ACC** | **PPC** |
| Metabolite FWHM (Hz) | 5.9 | 4.4 | 6.5 | 6.8 | 6.5 | 7.3 |
| Signal Noise Ratio (SNR) | 29.8 | 58.1 | 62.2 | 23.7 | 55.6 | 59.2 |
| Fit residual/noise ratio (Q) | 6.5 | 4.7 | 3.4 | 5.7 | 3.5 | 3.4 |
| Water FWHM (Hz) Tarquin | 7.2 | 7.1 | 7.5 | 8.4 | 8.5 | 6.8 |
| Water FWHM (Hz) Gannet | 7.9 | 8.8 | 7.5 | 8.7 | 8.8 | 9.0 |
